# Examining employment outcomes of deaf and hard-of-hearing people in The Netherlands using non-public microdata

**DOI:** 10.1177/10519815241289791

**Published:** 2024-11-13

**Authors:** Bas C.H.M. Elsendoorn, Loes Wauters, Corrie Tijsseling, Chris P.B.J. van Klaveren, Ilja Cornelisz, Eline C.M. Heppe

**Affiliations:** 1 Kentalis Academy, Royal Kentalis, Utrecht, the Netherlands; 2 Behavioural Science Institute, Radboud University, Nijmegen, the Netherlands; 3 Section of Methods and Statistics, Faculty of Behaviour and Movement Sciences, VU Amsterdam, Amsterdam, the Netherlands; 4 Amsterdam Center for Learning Analytics, Amsterdam, the Netherlands

**Keywords:** deafness, hearing loss, hearing impairment, employment, work, labor force, big data

## Abstract

**Background:**

It is internationally recognized that people with disabilities have an equal right to work as people without disabilities. This includes deaf and hard-of-hearing (DHH) people. Previous studies, primarily conducted in the US, showed discrepancies between employment outcomes of DHH people and typically hearing people.

**Objective:**

There is still a lack of knowledge about the current employment status of DHH people in the Netherlands. Furthermore, additional job characteristics such as income sources and sectors of employment are yet to be examined. This paper aims to address this lack of knowledge.

**Methods:**

This study used non-public microdata to examine employment outcomes of DHH people who claimed sensory disability care in the Netherlands between 2015 and 2019 (*n *= 5609) and compare them to a matched Dutch population (MDP) (*n *= 5609).

**Results:**

Despite small differences in employment participation, DHH claimants are more likely to have an income from sick leave/disability pension, other social benefits, and retirement compared to the MDP. DHH claimants also have lower average hourly wages, work fewer hours per week, work in different employment sectors, and stay in their first job longer compared to the MDP.

**Conclusions:**

The results of this study show that there are labor force differences between DHH claimants and the MDP. Further steps must be taken to identify the causes of these differences and develop policies and interventions to address these when deemed necessary.

## Introduction

For many adults, work is a vital aspect of life: not only does it ensure a standard of living for themselves and important people in their lives, but it also plays a central role in the development, expression, and maintenance of psychological health.^1,2^ Furthermore, work gives additional benefits such as structure to daily life, allows support from colleagues, regular supportive social engagement, and a sense of self-worth.^2^ While these are important, work can also offer more than these additional benefits of being employed: people who perceive their work as meaningful or as serving a greater social or community good, which has come to be known as meaningful work, also report better psychosocial adjustment and possess qualities that organizations find desirable.^3^ On the other hand, work that is deemed hazardous, stressful on a psychosocial level, or has negative elements affecting the organization of working life can have an adverse effect on employees, such as mental health problems, coronary heart disease, and musculoskeletal problems.^4,5^ Nevertheless, these adverse effects only occur under certain circumstances, and most of the time work positively impacts well-being and plays an important role in life.

The importance of work also applies to people with disabilities. Access to and the right to work for people with disabilities is acknowledged in Article 27 of the Convention on the Rights of Persons with Disabilities,^6,7^ which states that “States Parties recognize the right of persons with disabilities to work, on an equal basis with others.” As of January 2024, the Convention on the Rights of Persons with Disabilities had been ratified by 189 parties,^7^ signifying the international community's acknowledgment and importance of this Convention. Importantly, these treaties recognize that people with disabilities not only have the right to be employed but also to experience the same fulfillment from and benefits of meaningful work as people without disabilities. In the Netherlands, these rights were ratified in 2016.

### Employment outcomes of deaf and hard-of-hearing people

The right to work also applies to deaf and hard-of-hearing (DHH) people. DHH people face barriers when gaining employment and functioning in the workplace,^8^ including workplace discrimination.^9^ These barriers include communication difficulties, social, attitudinal, and structural barriers,^8–13^ and elevated stress and fatigue.^8^ A survey among 53 British deaf people showed that accessibility and inclusivity in the workplace is still a problem, with respondents reporting exclusion from conversations with colleagues (83%), loneliness at work (69%), exclusion from social events (59%), and bullying and unkindness at work (34%).^14^ Despite these barriers, a qualitative study using focus groups found that people who became deaf at an early age do express a desire to work.^12,15^ Using an online questionnaire, Houston and colleagues^16^ found that professionals who provide employment-related services for DHH people agreed or strongly agreed with the statement that there is an increased number of DHH clients seeking help to find employment, suggesting that there might be an increased desire in DHH people to find work.

We recognize that there are many ways to describe the hearing status of DHH people, with terms that were considered the norm now being outdated and offensive. The authors aim to write about DHH people in an inclusive manner, focusing on terms coined under the umbrella of functional diversity.^17^ Nevertheless, while we have tried to replace outdated designations (such as “hearing impairment”) with more inclusive terms, we recognize that some common words are not inclusive and have no suitable replacement. One example would be hearing loss: although this could refer to a reduction in the degree of hearing of a person, it can also imply a “loss” of capabilities and worth of a person. Nevertheless, being DHH can also come with gains, such as skills that typically hearing people do not commonly possess, for example, greater development of peripheral vision.^18,19^ We have tried to avoid offensive terminology wherever possible.

#### Employment participation

Previous scientific research has found differences in employment participation between DHH people and typically hearing people. In their systematic review of studies published before 2018, Shan and colleagues^20^ found only a few high-quality studies on employment in the DHH population. Nevertheless, these studies all reported a higher likelihood of unemployment in DHH individuals. In a more recent survey study, Garberoglio and colleagues^21^ found that, in the US in 2017, among those who reported being deaf or having serious difficulty hearing (*n* ≈ 37.700), 53% were employed, compared to 76% of the US population. In a Danish survey study, Dammeyer and colleagues^22^ found that 38% of 804 DHH individuals (self-reported hearing loss between 45 dB and > 91 dB) aged between 16 and 65 years were employed, compared to 67% of a randomly selected Danish reference group. In this study, individuals on sick leave were not counted in the employed category (33% of the DHH individuals and 13% in the reference group). Using interviews and questionnaires, Emmett and Francis^23^ found that US adults aged 20–69 with hearing loss (defined according to the World Health Organization criteria) had nearly two times higher odds of being unemployed (95% confidence interval, 1.38–2.85). In Sweden, of 2144 deaf people (defined as those who had attended Swedish primary or secondary deaf education), 63% were in employment, compared to 78% of a randomly selected reference population (n = 100,000).^24^ In the Netherlands, Stam and colleagues^25^ found that 61% of 892 participants with hearing loss (defined using questionnaires and an online speech-in-noise test) were employed, compared to 68% of 996 typically hearing people. Thus, it appears that those who experience difficulty in hearing are less likely to be employed compared to typically hearing people; however, the extent differs across studies and nations.

The participation of DHH people in the labor force can mutually benefit them and their employers. DHH people report that being employed gives them financial security, a sense of purpose and well-being, and it prevents them from having mental health problems.^15^ From the employer's perspective, a systematic review found that deaf people had excellent work habits and a strong work ethic, with performance beyond job functions.^26^ This mutual benefit for both employee and employer underscores the importance of making the labor market accessible to DHH people. To do so, however, it is necessary to focus not only on employment participation but also on job characteristics.

#### Job characteristics

Some studies including DHH people have also found differences in job characteristics, such as sectors of employment, hourly wages, and weekly working hours. Looking at sectors of employment, Garberoglio and colleagues^21^ found that the most common sector in which DHH people worked in the US was manufacturing. For typically hearing people, the most common sector was the medical industry. The same study also found that DHH people and typically hearing people earn similar median annual wages (USD 50,000 and USD 49,900, respectively); however, median annual earnings varied widely between the two groups based on race and the presence of additional disabilities (such as blindness, cognitive disabilities).^21^ This study^21^ also found that DHH people were more often self-employed compared to typically hearing people.

Dammeyer and colleagues^22^ categorized the current employment of participants into four categories and found that 4% of the DHH individuals had their own company, 9% were in employment with management responsibility, and 25% without management responsibility. This compared to 9%, 15%, and 43%, respectively, in the reference group. Dong and colleagues^11^ categorized the employment of 59 US DHH individuals (self-reported DHH) in terms of the level of management involved in the job and found that 41% had non-managerial positions, 25% had lower level managerial positions, 29% middle level, and 5% upper level. They^11^ also found that 70% of the DHH individuals in their study worked full-time. These results were not compared to a US reference group.

Stam and colleagues^25^ focused on working hours and monthly income, finding that people with hearing loss worked a mean of 30 hours per week, while typically hearing people worked 32 hours per week. For lower educational levels, lower hearing ability was also associated with lower levels of monthly income.^25^ These studies all demonstrated differences in job characteristics between DHH people and typically hearing people. However, some of these job characteristics have not yet been studied in DHH people in the Netherlands nor compared to the general Dutch population.

### Methodological challenges in studying employment outcomes of deaf and hard-of-hearing people

It is worth noting that the studies described above used different methods to define DHH when determining their DHH inclusion criteria, including self-reports,^11,21,22^ education characteristics,^24^ and audiometric tests.^23,25^ We would like to acknowledge that the DHH population is diverse and that different definitions of DHH are valid in determining study participants; however, this also means that it is likely that the employment status of different subgroups of DHH people has been studied. In other words, most studies have focused on and included only one specific subgroup. This probably also partly explains differences in employment outcomes for DHH people across studies, as variation in hearing status may be associated with level of employment participation.

Furthermore, research on employment outcomes of DHH people is often undertaken using surveys^10,11,14,21,25^ or qualitative interviews.^15,16,27^ While these methods are valuable for understanding the daily experiences and challenges faced by DHH people in the labor force, they are less valuable for analyzing employment outcomes. Also, the sample sizes of the studies vary greatly. This makes it difficult to generalize the findings to the entire population, especially since the DHH population is heterogeneous, with the onset of deafness, its severity, and adjustment to hearing status varying greatly from person to person, even when they have identical audiometric test results.^28^ Therefore, examining employment outcomes on a societal level using automatically generated administrative data (a type of big data) might better suit the aim of objectively determining the current position of DHH people in the labor market.

### Using big data to examine employment outcomes of deaf and hard-of-hearing people

Gaining detailed insight into employment outcomes using big data aims to address the first step in the 5D model: a theoretical framework designed to improve policy interventions.^29^ The model consists of five phases: detect, diagnose, design, determine, and decide. Thus, the scope of this paper concerns the detect phase of the 5D model, which identifies potential problems and provides detailed insight into what needs to be addressed and resolved in the subsequent steps of the 5D model.^29^ One type of big data that can help detect these problems is automatically generated administrative data, also called microdata. Microdata has been used since the 1950s^30^ and, more specifically, has been used successfully to examine employment outcomes in various studies, such as market polarization,^31^ wage growth,^32^ and job characteristics.^21^ Microdata has also been used to estimate the prevalence of intellectual disabilities in the Netherlands,^33^ but as far as the authors are aware it has not been used to examine employment outcomes of DHH people in the Netherlands.

Microdata can be used to study particular communities^30^ and is therefore helpful for studying a specific population, such as DHH people. Microdata consists of large amounts of objective records describing individuals^30^ and, combined with new technologies to analyze such data, can provide an accurate overview of the employment status of DHH people in the Netherlands. The use of microdata avoids potential bias that might arise due to the selective inclusion of DHH people and avoids common barriers found in studies using surveys or semi-structured interviews, such as socially desirable answers.^34^ Moreover, microdata does not require active recruitment of participants and thus avoids exclusion based on language choice, since some DHH people prefer sign language and others prefer spoken language. In short, the use of microdata may complement current research and provide a more comprehensive picture of the employment outcomes of DHH people in the Netherlands.

### Current study

The present study aims to examine employment outcomes of DHH people in the Netherlands and compare the findings to a matched Dutch population (MDP) using non-public microdata. The MDP is a control group constructed from the Dutch population with similar background characteristics to the DHH people in the study. The results of this study should provide a more representative view of the labor force status of Dutch DHH people. To achieve these aims, this paper answers two research questions: 1) What is the *employment participation* (percentage of employed people and income sources) of DHH people who claimed sensory disability care in the Netherlands between 2015 and 2019, and is it comparable to that of the MDP?; 2) What are the *job characteristics* (hourly wages, average working hours, sectors of employment, length of employment) of DHH people who have a job and claimed sensory disability care in the Netherlands between 2015 and 2019, and are these comparable to those in the MDP who have a job?

## Methods

### Design and data sources

This study uses non-public microdata compiled from administrative data from various organizations (such as municipalities and insurance companies) held by Statistics Netherlands (SN). This data is available for statistical and scientific research upon request under strict conditions. Due to changes in Dutch policy and legislation and the periodic release of data by SN, only data on claims for Dutch sensory disability care between 2015 and 2019 could be used in this study. Formal ethical approval was not requested for this study, as under Dutch privacy law ethical approval is not required when the results are not traceable to an individual level.^35^ For this study, four datasets were used: the first contained basic demographic information such as age, gender, and migration background; the second contained claims made by people who used sensory disabled care in the Netherlands between 2015 and 2019; the third contained information regarding the socioeconomic status of the individual based on several income sources; and the fourth contained data on job characteristics such as hourly wages, average working hours, sector of employment, and length of employment.

### Study population

This study included 5609 DHH people who claimed sensory disability care in the Netherlands between 2015 and 2019. Sensory disability care can be claimed by people with visual impairment, people with developmental language disorder, and DHH people. For this study, we only used data from DHH people. For the sake of brevity, we will refer to this DHH population as “DHH claimants.” People using sensory disability care in the Netherlands must meet the evidence-based guidelines established by the Federation of Dutch Audiological Centers.^36^ The definition of DHH in these guidelines aligns with the classification of hearing loss by the World Health Organization.^37^ More specifically, people with a moderate hearing loss of 35 decibels or more in their better ear, according to the Fletcher Index high qualify for sensory disability care.^36^

People who are deaf or hard-of-hearing and eligible for sensory disability care in the Netherlands are entitled to interventions that focus on coping strategies to alleviate the mental burden of their disability, as well as interventions aimed at reducing the disability itself, thereby enhancing self-sufficiency.^36^ Examples include learning Dutch Sign Language, communication training for people who become deaf at an older age, and therapies for learning social skills. It does *not* include complex, long-term, and life-encompassing care, care and support to function in society, medical care, psychiatric care, provision of sign language interpreters, audiometric tests, or support related to hearing aids and cochlear implants.^36^

As shown in Table 1, the average age of the DHH claimants was 29 years (ranging from 0 to 98 years; SD = 26.34), 52% were registered as female, 12% were considered a first-generation immigrant (as defined by SN), and 20% were considered a second-generation immigrant (as defined by SN). Explorative data analyses showed differences between DHH claimants and the general Dutch population in age, gender, highest level of education achieved, and migration background. Previous studies have shown that these factors are related to labor force outcomes.^21,24,25^

**Table 1.** Background characteristics and highest achieved education of the DHH claimants.

Background characteristics	DHH claimants (n = 5.609)
Average age (years)	29
Gender (female)	52%
1^st^ generation immigrant	12%
2^nd^ generation immigrant	20%
Highest achieved education	DHH claimants (n = 5.609)
No information	19%
Primary education	58%
Pre-vocational secondary education^a^/first level secondary vocational education^b^/first three years of senior general secondary education^c^ or pre-university education^d^	7%
Second to fourth levels of secondary vocational education/final years of senior general secondary education or pre-university education	10%
Master's degree of higher professional education^e^ or university	6%

^a^ In Dutch: Voorbereidend middelbaar beroepsonderwijs (vmbo).

^b^ In Dutch: Middelbaar beroepsonderwijs (mbo).

^c^ In Dutch: Hoger algemeen voortgezet onderwijs (havo).

^d^ In Dutch: Voorbereidend wetenschappelijk onderwijs (vwo).

^e^ In Dutch: Hoger beroepsonderwijs (hbo).

To account for these differences, the DHH claimants were matched with the general Dutch population on age, gender, highest level of education achieved, and migration background. This population is referred to below as the “matched Dutch population” (MDP). The background characteristics of the MDP are thus similar to the characteristics of the DHH claimants due to this matching process. It should be noted that DHH people may also occur in the MDP, but importantly, none of the MDP individuals claimed sensory disability care in the Netherlands between 2015 and 2019. The matching procedure resulted in one drop-out, as no match was found for one individual. Both groups resulted in a total sample size of 11,218 people: 5609 DHH claimants and an MDP of 5609 people.

### Employment outcomes

The primary outcome (first research question) of this study was employment participation, defined by the *percentage of employed people* and *income sources*. SN defines employment as “a professional employee-employer relationship and at least one hour of paid labor conducted in a week”.^38^ Regarding income sources, SN defines 13 different income sources. The definitions of these income sources as provided by SN are shown in Table 2. The primary outcome “employment participation” is thus examined through two different datasets, which could lead to different results. The secondary outcome of this study (second research question) was job characteristics. This study included four job characteristics: *average hourly wages*, *average working hours*, *sectors of employment*, and *average length of employment*. The definitions provided by SN for these job characteristics are shown in Table 2.^39^

**Table 2.** Income sources of the DHH claimants and the MDP between 15 and 65 years of age and definitions of the income sources and job characteristics.

Income sources	DHH claimants (n = 2.236)	MDP (n = 2.236)	Definition
Employee	77%	83%	A person who gains income as an employee in the Netherlands or from labour abroad, without having a position as director or shareholder
Director/Shareholder	1%	2%	A person who has a primary job as a director/shareholder
Self-employed	6%	13%	A person who gains income out of a self-owned business
Other self-employment	11%	13%	A person who gains income from other self-owned businesses
Unemployment benefits	25%	27%	A person who gains income via governing laws defining unemployment benefits
Social security	33%	34%	A person who gains income via governing laws defining social security
Other social benefits	44%	28%	A person who gains social benefits not via governing laws defining social security. Examples include people with early-onset disabilities starting from childhood, veterans and artists.
Sick leave/disability pension	49%	29%	A person who gains income via governing laws defining sick pay and disability pensions
Retirement	28%	19%	A person who gains income out of pension or retirement funds.
Pre-school/attending school/studying with income	5%	5%	A person who is a student with a monthly income.
Pre-school/attending school/studying without income	50%	49%	A person who is a student without a monthly income.
Others categories without income	41%	40%	A person without income without attending school.
Employed family member	40%	50%	A person who are a partner in the business of those with self-employment as income source.
Job characteristics	Definition		
Average hours of work per week	The contract monthly hours as recorded in data for income taxes divided by 4.325 (the average number of weeks per month).		
Hourly wage	Monthly wage divided by monthly hours both recorded in data and income taxes.		
Fields of employment^a^	The sector which an employee is working in.		
Average length of first job	The average length of the first job held by a person as defined in months.		

^a^ The fields of employment are based on the sector affiliation as determined by the Dutch Tax and Customs Administration.^40^

### Statistical analysis

#### Analysis: primary and secondary outcomes

To answer the first research question, DHH claimants were compared to the MDP based on the proportion of those with a paid job at any point during 2015–2019. In addition, the relative prevalence of the different income sources observed in this period for DHH claimants was compared to the MDP and these comparisons were disaggregated by age for income sources.

To answer the second research question, the subgroup of working DHH claimants was compared to their working counterparts in the MDP on average hourly wages by age, average working hours per week, sectors of employment, and average length of employment (first job and working career average). Finally, we ranked the ten most common sectors of employment for working DHH claimants and compared these ranks to those observed for these sectors in the MDP.

#### Analysis: matching

The MDP is a control group constructed from the Dutch population with similar mean background characteristics (i.e., gender, age, migration background, and highest education level achieved). Therefore, these background characteristics will not drive outcome differences between the DHH and MDP. To construct the MDP, a propensity score matching procedure was used, with a nearest neighbor matching algorithm and a 0.005 caliper distance measure. A one-sample t-test was used to test whether the mean differences between the MDP and the DHH claimants was statistically significant. A difference was considered significant if the population mean of the DHH claimants lay outside the 95% confidence interval of the sample mean of the MDP. The differences between the MDP and DHH claimants presented in this paper were all statistically significant.

## Results

### Primary outcome: employment participation

To study the primary outcome of the study, only people aged between 15 and 65 years of age (working age population) were included (n = 2236). Due to the matching procedure, both populations (DHH claimants and MDP) comprised the same number of people in that age group.

#### Percentage of employed people

The percentage of employed people between 15 and 65 years of age (n = 2236) for DHH claimants was 70%, while for the MDP it was 75%.

#### Income sources

Table 2 shows the income sources for DHH claimants and the MDP. The largest income source for DHH claimants between 15 and 65 years of age was “employee,” at 77%, and the smallest income source was “director/shareholder,” at 1%. For the MDP, the largest and smallest income sources were also “employee” and “director/shareholder,” at 83% and 2%, respectively. Although the differences between income sources for DHH claimants and the MDP were small in most categories, there were some notable differences. The largest difference was found for the income source “sick leave/disability pension,” with 20% more DHH claimants having sick leave/disability pension as an income source compared to the MDP. Other notable differences in income source between DHH claimants and the MDP included the category of “other social benefits” (16% difference) and the category of “retirement” (11% difference). Finally, fewer DHH people between 15 and 65 years of age had “self-employment” as an income source compared to the MDP.

### Secondary outcome: job characteristics

Only working people aged between 15 and 65 years of age were included in the study of the secondary outcome.

#### Average hourly wages

**Figure 1.**
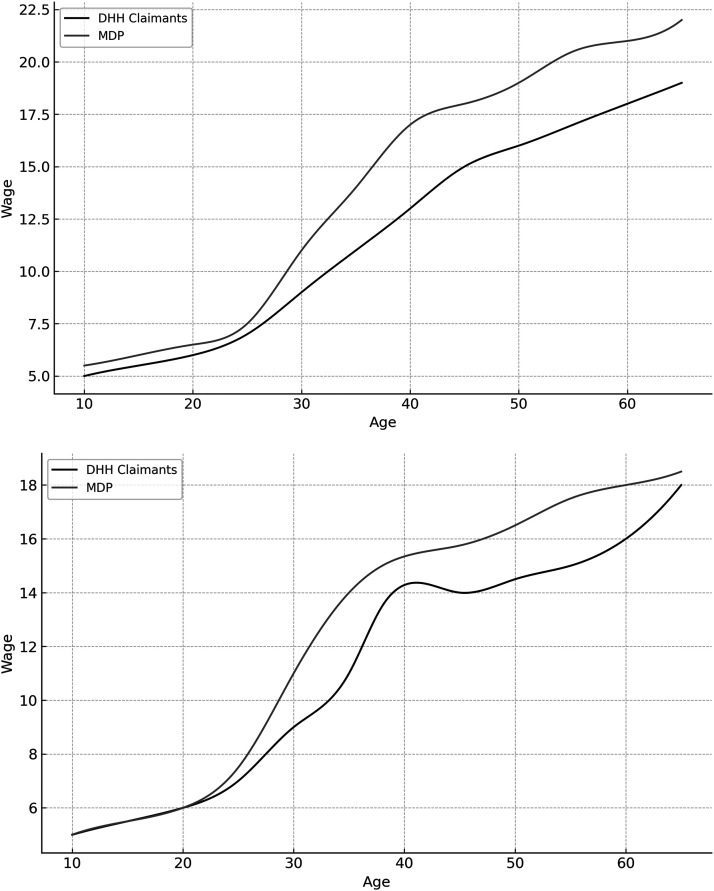
Average hourly wages for the DHH claimants and the MDP between 15 and 65 years of age *(men: upper panel, women: lower panel)*.

Figure 1 shows that for male DHH claimants between 15 and 65 years of age, there is a steady increase in average hourly wage over time. For female DHH claimants between 15 and 65 years of age, there is a steady increase in the average hourly wage until 35 years of age. After a period of stabilization, the average hourly wage starts steadily increasing again for female DHH claimants around the age of 55, until it reaches a peak around 65 years of age. Figure 1 also shows that a difference in average hourly wage between DHH claimants and the MDP was found for both genders, which increases around the age of 25 years. It is also apparent that both male DHH claimants and female DHH claimants earn less than their counterparts in the MDP. For DHH claimants of both genders, differences in hourly wage compared to the MDP are most prominent around the age of 50.

#### Average working hours

**Figure 2.**
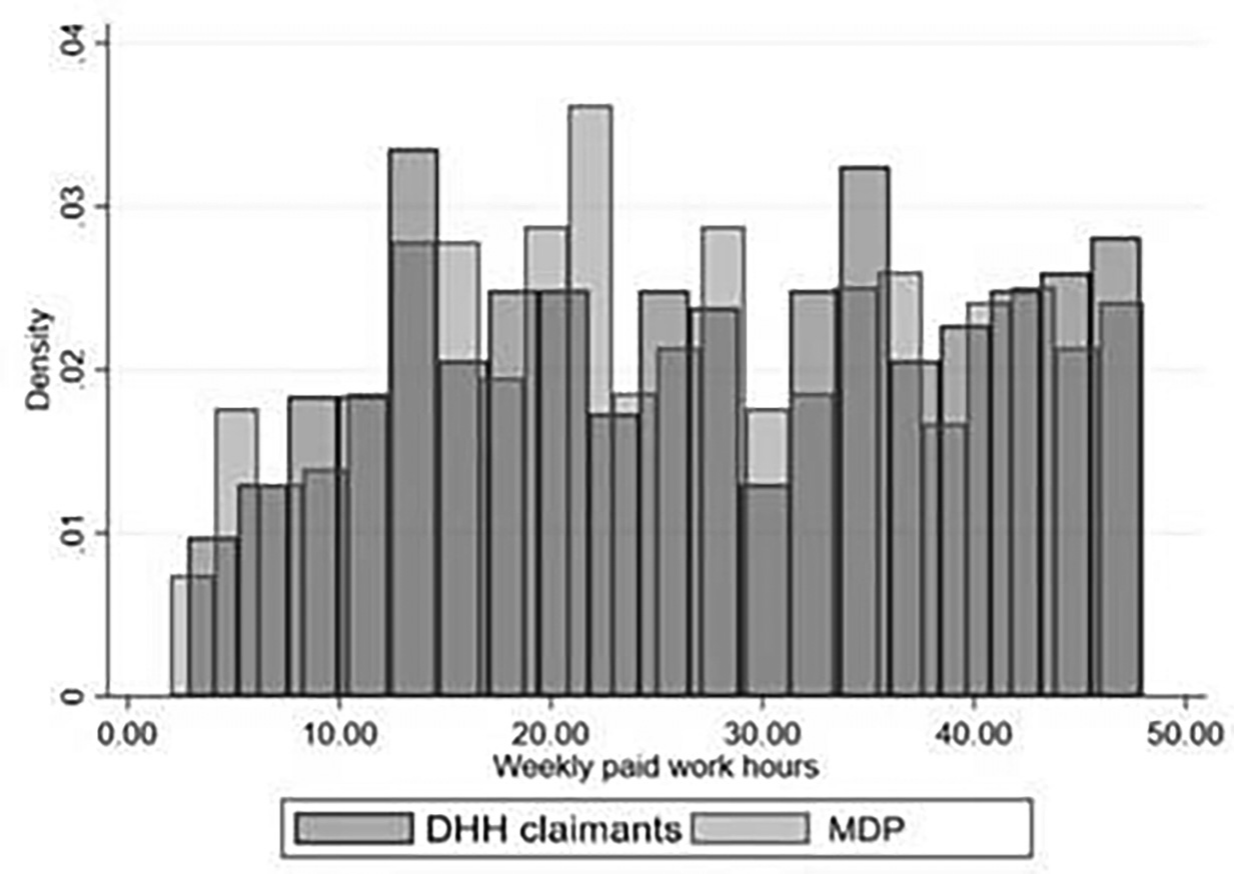
Average working hours per week for the DHH claimants and the MDP.

Figure 2 shows the average weekly working hours for the DHH claimants and the MDP. The graph shows a peak for DHH claimants at 14 paid hours per week, while for the MDP population, this is 22 hours per week. Furthermore, DHH claimants more often work 36 hours per week compared to the MDP, who more often work 38 hours per week. Overall, the density in average working hours is generally evenly distributed for DHH claimants and the MDP.

#### Sectors of employment

Table 3 shows that the three most common sectors of employment for DHH claimants are in the physical and mental health, education, and social interests sector, followed by temporary job agencies, the work and (re)-integration sector, the hospitality industry, and in government, education, and science organizations, respectively. Table 3 also shows that the sectors of employment for DHH claimants are largely similar to the MDP, with two exceptions. The work and (re)integration sector is the third largest sector of employment for DHH claimants, while it is only the thirty-first largest sector of employment for the MDP. The work and (re)integration sector comprises institutions and services that are charged with the implementation of the law concerning social services and the obligations of job seekers: this means that these institutions and services are responsible for reducing the reliance on social security/welfare and assisting job seekers to find suitable work. Additionally, government, the provinces, municipalities, and regional water authorities combined are the tenth largest sector of employment for DHH claimants, in contrast to being the sixteenth largest sector of employment for the MDP. The jobs in this sector mainly consist of administrative duties.

**Table 3.** Top 10 sectors of employment for the DHH claimants and the MDP.

DHH claimant ranking		MDP ranking
1.	Physical and mental health, education and social interests	1
2.	Temporary job agencies	2
3.	Work and (re)-integration	31
4.	Hospitality industry	3
5.	Government, education, and science	8
6.	Department stores, chain stores, and big-box retailers	7
7.	IT, engineering, and marketing firms	5
8.	Retail and real estate	4
9.	Intermediaries, journalism, collection agencies, and interpreters	6
10.	Government, provinces, municipalities, and water authorities	16

#### Average length of employment

Regarding the average length of employment, DHH claimants remain in their first job for 1258 days on average (*SD *= 1316.45) and for 1171 days on average (*SD *= 1216.38) in the same position over their entire career. The MDP remain in their first job for 1175 days on average (*SD *= 1294.52) and 1269 days on average (*SD *= 1153.31) in the same position over their entire career. Overall, these results show that DHH claimants stay in their first job longer compared to the MDP but, on average, they are employed for a shorter period over their entire career.

## Discussion

In this study, employment outcomes (employment participation and job characteristics) of DHH claimants who claimed sensory disability care in the Netherlands between 2015 and 2019 were compared to an MDP using non-public microdata. A small difference was found between the percentage of employed DHH claimants compared to the MDP, which is consistent with findings in previous studies on employment participation.^21–25^We also found that DHH claimants were more likely to have an income from sick leave/disability pension, other social benefits, or be retired compared to the MDP. While Garberoglio and colleagues^21^ found that DHH people were more often self-employed compared to typically hearing people, the reverse was found in the current study, supporting the findings of the study by Dammeyer and colleagues.^22^ Furthermore, on average, DHH claimants had a lower hourly wage compared to the MDP (this was the case both for DHH male and female claimants). Although differences in working hours per week between DHH claimants and the MDP were relatively small, DHH claimants were more likely to work 36 hours per week and the MDP 38 hours per week. The finding that Dutch DHH people are more likely to work fewer hours compared to typically hearing people aligns with the results of the study conducted by Stam and colleagues.^25^ Furthermore, DHH claimants more often worked in the work and (re)-integration sector and in government, province, municipality, and water authorities, compared to the MDP. Finally, DHH claimants remained longer with their first employer compared to the MDP.

These results show that to determine whether employment outcomes of DHH people are truly equal to a general population, it is worth looking beyond the binary “yes/no” question of whether someone is employed or not. Although at first glance there appears to be a relatively small difference in employment participation between DHH claimants and the MDP, our secondary outcomes show that this is not the case for job characteristics; for example, the lower hourly wage could potentially be an indicator that DHH claimants are not receiving equal pay for their work. Although examining the causes of the differences between average weekly hours and sectors of employment is beyond the scope of this study, the question arises as to why these differences exist. Possible explanations may be that facilities for DHH claimants are inadequate for full-time work; different sectors of employment may have a lack of accessibility to specific subsectors of work; and/or there may be factors driving DHH claimants to work in specific sectors of employment. Finally, although a longer average length of stay in the first job of DHH claimants may be preferable because of general satisfaction with the job or the job security offered, it might also stem from a reluctance to change jobs because of stigma from potential new employers, which in turn prevents flexibility throughout the course of DHH claimants’ careers.

Consistent with the first step of the 5D model (the “detect” stage), the results of the current study only investigated whether there were differences in employment outcomes between DHH claimants and the MDP, which we found. To further address these disparities, studies that take the next steps in the 5D model should also be undertaken. There is a need to: determine the causes of these differences and who would benefit from potential interventions that address these disparities (diagnose); design the intervention (design); evaluate whether the proposed intervention is successful through causal evaluations (determine); and, finally, carefully interpret the findings and successfully translate them into sustainable changes in practice and policy (decide). All these steps must be made carefully and deliberately, as haste can affect the success of the implementation of interventions and policies that aim to address disparities.^29^

### Study limitations

This study has some limitations. First, data are currently only available for DHH people who claimed sensory disability care in the Netherlands between 2015 and 2019. This is due to a change in the law in the Netherlands that revamped the healthcare system. Another limitation of the study is that the data only included those DHH people who claimed sensory disability care. While this resulted in a large sample, this group is specific in terms of the requirements for people to be able to claim such care. To be included in the data, DHH people had to have a health question related to their sensory disability, and they had to find their way through the Dutch healthcare system to have these questions addressed. Other Dutch DHH people who did not use sensory disabled care in the Netherlands between 2015 and 2019 were thus not included in the sample. These DHH people may use sign language interpreters, hearing aids, cochlear implants, and other forms of provision or care that are not included in sensory disability care. Thus, because data was unavailable for DHH people who did not receive sensory disabled care (due to choice, lack of accessibility, insufficient knowledge about the possibilities for this type of care, having claimed sensory disability care before 2015, or other reasons) this study has potentially overlooked a group of DHH people. While the results found for DHH claimants in this study may be an indicator for other subgroups of the DHH population, we cannot generalize the results of the current study to the entire Dutch DHH population.

There are many advantages to using non-public microdata, but there are also limitations to using this type of data. For example, this study was ultimately limited to the definitions set by SN for the data, which may result in datasets that do not provide a complete view of whether work opportunities are genuinely equal for DHH claimants. For example, the data could not show subjective variables such as whether DHH claimants were satisfied with their current work, which is an important factor in measuring whether work is meaningful.^3^ In line with the 5D model, these subjective variables should be examined in the “diagnose” phase before proceeding to the “design” phase.^29^ For example, concerning the lower hourly wage found in this study, it should be examined why specific subgroups of DHH claimants have lower hourly wages and whether they are satisfied with their hourly wage. The diagnose phase requires attention before turning to the next step of designing interventions or policies addressing these lower wages.

### Implications of results

The differences found in this study may have several implications for advocacy groups and policymakers. This study offers greater societal awareness of the position of DHH claimants in the Dutch labor market. Advocacy groups for DHH people can use the results of this study to call upon future research in line with the steps of the 5D model.^29^ Implications for healthcare and education may also be identified in future research, but only after addressing the other steps in the 5D model.

Although this article addresses employment outcomes of DHH claimants in the Netherlands and the results cannot be generalized to the total Dutch DHH population, to DHH populations in other countries, or to the global DHH population, these results may still be valuable for international DHH advocacy groups seeking to raise awareness of this issue in their own country. As a significant number of countries in the European Union have ratified the Convention on the Rights of Persons with Disabilities,^7^ these countries have committed to improving equality in their countries, including the right to equal work for DHH people. This study contributes to this commitment by making other countries and communities aware that DHH people may still face inequalities and allowing comparison of the equality standards of different communities and nations.

In addition, this study may inform researchers in other countries about the opportunities that non-public microdata offer for examining the labor market status of DHH people: in particular, the methodology of this study can provide a blueprint for more standardized methods of examining employment participation of the DHH population and other specific populations.

### Future research

We recognize that SN (and other institutions with similar functions) will continue to collect non-public microdata from DHH claimants in the coming years. These datasets may include DHH people who were not included in the current study, due to the passage of time and new data from other sources, such as other forms of claimed care. Additionally, agencies dealing with employment for the Dutch population (including DHH people) may include their data in future datasets, such as the Dutch Employees Insurance Agency and reintegration services. All these additional sources may lead to a more complete picture of the current status of DHH people in the Dutch labor force. Moreover, due to the dynamic nature of the economy and the increasingly flexible nature of jobs and employment, it is important that future studies ensure they stay abreast of the current employment status of DHH claimants and the outcomes used to measure whether their employment opportunities are equal to the general population.

Research on the employment outcomes of other populations with disabilities may also be worthwhile. As stated in the Convention on the Rights of People with Disabilities,^6^ equal work applies to everyone with a disability. For the current DHH claimant group, research at the detect phase in the 5D model has started, but for other groups with disabilities, this has yet to be done. Examples of these groups include other populations of claimants within the Dutch sensory disability care system, such as people with a visual impairment and people with a developmental language disorder. Beyond the population of people with sensory disabilities, the topic of employment is also worth exploring in other groups of people with disabilities, such as people with physical disabilities (e.g., those using a wheelchair) and those with neurological diversity. The identification of potential inequalities in the labor force for all people with disabilities deserves more attention. Moreover, while there are differences among people with disabilities, some reasons for differences in their labor force status compared to people without disabilities may be shared. When the causes of inequality are detected and diagnosed, potential interventions and policies may benefit multiple populations with disabilities.

## Conclusion

This study provides insight into employment outcomes of DHH people who claimed sensory disability care in the Netherlands between 2015 and 2019. To the authors’ knowledge, this is the first paper to examine income sources, hourly wages, sectors of employment, and length of employment among DHH claimants in the Netherlands. Furthermore, to the authors’ knowledge, this is the largest sample of Dutch DHH people examined on employment participation and job characteristics, giving us a more complete picture of the labor force status of DHH claimants in the Netherlands.

The results highlight that there are differences in employment outcomes between DHH claimants and the MDP. The results of this study can be used to diagnose the causes of these differences and determine future steps to address these differences. These insights will potentially support the development of future policies and interventions to improve the employment outcomes of DHH people in the Netherlands.
